# The Impact of COVID-19 on Medical Device Reporting and Investigation

**DOI:** 10.33940/data/2021.9.3

**Published:** 2021-09-17

**Authors:** Zoe Pruitt, Christian Boxley, Seth A. Krevat, Srijan Sengupta, Raj M. Ratwani, Allan Fong

**Affiliations:** ◇ MedStar Health National Center for Human Factors in Healthcare; ‡ Georgetown University School of Medicine; § North Carolina State University

**Keywords:** COVID-19, MAUDE database, medical device reporting

## Abstract

**Introduction:**

The Manufacturer and User Facility Device Experience (MAUDE) database houses medical device reports submitted to the U.S. Food and Drug Administration (FDA). In May 2020, the FDA released guidance about medical device reporting during a pandemic, anticipating delays in reporting and investigating events involving medical devices.

**Methods:**

We aimed to understand how the COVID-19 pandemic impacted medical device reporting by analyzing reports in the MAUDE database that mention COVID-19.

**Results:**

From the 816,470 reports submitted between January 1 and July 31, 2020, 3,500 (0.43%) included phrases related to COVID-19. Of these reports, 4.8% (167/3,500) described adverse events during COVID-19 patients’ treatment, and 90.3% (3,161 /3,500) described barriers manufacturers faced investigating malfunctioning devices during the pandemic. 4.9% (172/3,500) of reports were not related to COVID-19. Malfunctions were clinically significant in 85.8% (3,004/3,500) of reports.

**Discussion:**

Reports indicate challenges some manufacturers had when investigating medical devices during the pandemic. The pandemic made investigating implants uniquely difficult, as restrictions to person-to-person contact limited the type of care patients could receive. Because full-scale investigations into malfunctioning devices may be difficult to perform during the pandemic, safety issues may go unaddressed and result in future harm to patients.

**Conclusion:**

The COVID-19 pandemic and the myriad of healthcare, travel, and shipping challenges it created impacted how manufacturers reported and investigated medical devices. At the current time, it is unclear how manufacturers will address delayed clinical management of implant devices and other uninvestigated malfunctions after the pandemic and how this will impact patient safety.

COVID-19 and the resulting pandemic changed nearly every aspect of medicine, including medical device reporting. Medical device reporting is a strategy used by the U.S. Food and Drug Administration (FDA) to monitor medical device safety.^1^ The FDA stores submitted reports in the Manufacturer and User Facility Device Experience (MAUDE) database, which serves as a passive tool for postmarket surveillance.^2,3^ Anyone can submit reports voluntarily, but some entities (e.g., manufacturers, importers, user facilities) are mandatory reporters and must report adverse events.^4^

The FDA asks mandated reporters to submit reports within a specific time frame, typically 30 days for manufacturers.^5^ However, in May 2020, the FDA released updated guidance outlining the government agency’s expectations for adverse event reporting during a pandemic.^6^ The guidance requests that firms report adverse events when possible but that the “FDA does not intend to object” if reports are delayed due to pandemic-related “high employee absenteeism.”

It is evident that the FDA expected the pandemic to disrupt manufacturer’s overall capabilities to report issues with medical devices. However, it remains unclear exactly which aspects of the reporting and investigative process have been impacted by the pandemic. The goal of this paper is to answer this question. The pandemic presents a multitude of unique factors to the healthcare system and the world, which could impact medical device reporting. These include increased use of specific devices (e.g., ventilators), the moratoriums on elective procedures, patients’ reluctance to seek in-person care, limited travel, and delayed postal service.

MAUDE contains millions of reports about medical devices, and some of these reports include data about the efficacy and safety of medical devices. However, it is difficult to find relevant and actionable signals in the noise. We sought to analyze reports in the MAUDE database that mention COVID-19 or related terms. We used word search and manual review to understand how the COVID-19 pandemic impacted medical device reporting. The resulting reports describe manufacturers’ challenges to investigate medical device events due to the pandemic.

## Methods

Reports submitted between January 1, 2020, and July 31, 2020, were retrieved from the MAUDE database via keyword search. The search period begins in January because this is when COVID-19 was first identified in the United States.^7^ Reports were included in the manual review if they contained COVID-19 phrases (“covid,” “corona,” “coronavirus,” “covid-19,” “covid19,” “pandemic”) in either the free-text description entered by the reporter or the manufacturing narrative entered by the device manufacturer. The reports with COVID-19-related phrases were categorized using the code-book described in Table 1.

Two coders (ZP, CB) dually coded 10% of the data with the code-book. Coders discussed disagreements when necessary, and a third coder (AF) was included as a tiebreaker when necessary. The inter-rater reliability kappa scores were 1.0 for Relevance to COVID-19 and 0.85 for Patient Care Impact.

## Results

**Figure 1.**
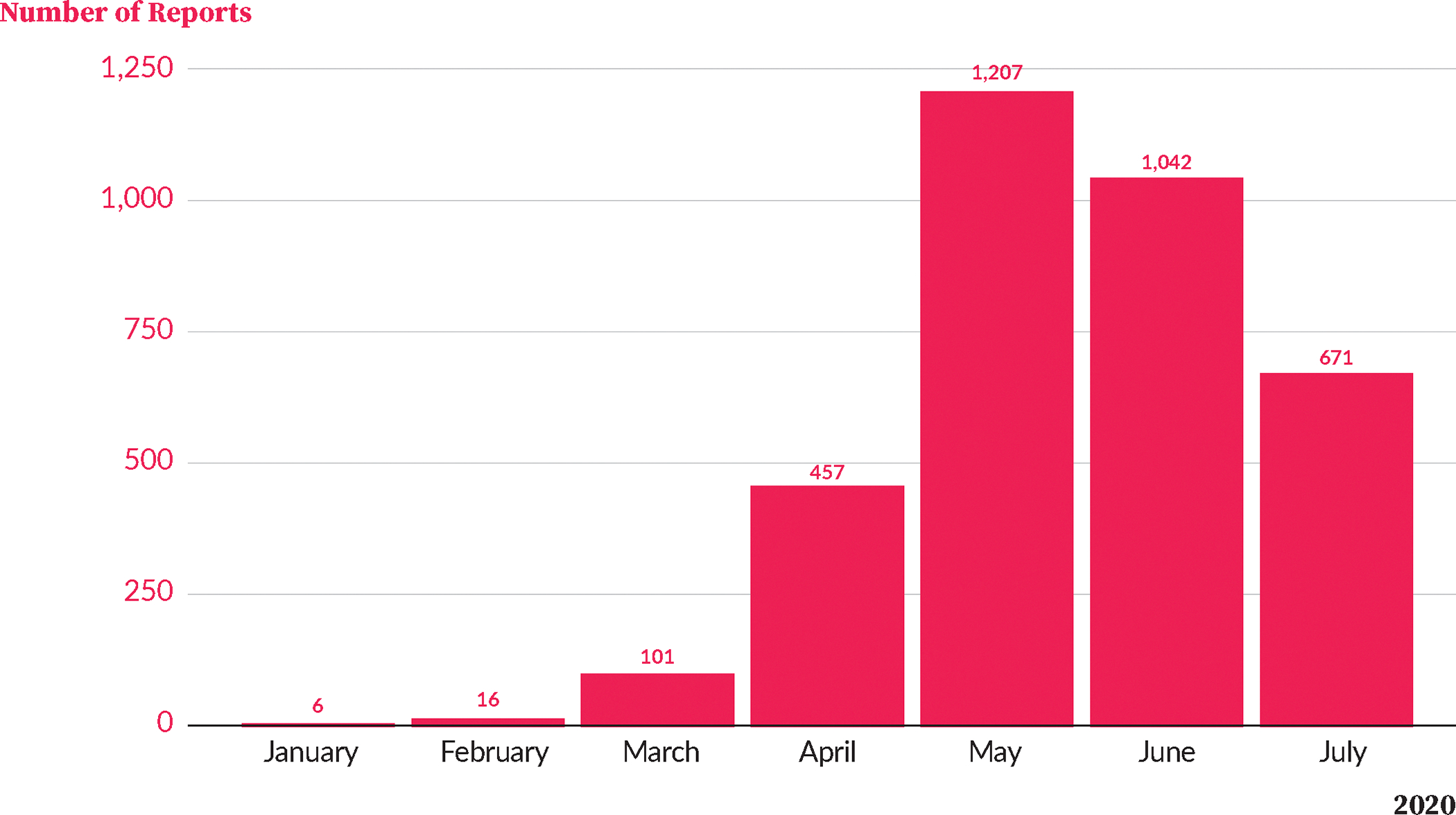
Frequency of MAUDE Reports With COVID-19-Related Phrases Between January 1, 2020, and July 31, 2020

From the 816,470 MAUDE reports, 3,500 (0.43%) included COVID-19 phrases, of which 167 (4.8% of 3,500) were coded as COVID-19 Treatment, 3,161 (90.3% of 3500) were coded as Investigative Delay, and 172 (4.9% of 3500) were unrelated to COVID-19. See Table 2 for details. Reports that included COVID-19 phrases peaked in May 2020, with 1,207 (34.5%) reports. The distribution of reports over time can be found in Figure 1.

### Reports Coded as COVID-19 Treatment

Of the 167 reports coded as COVID-19 Treatment, 122 (73.1%) related to COVID-19 tests or COVID-19 antibody tests, 32 (19.2%) described issues with equipment used in the treatment of COVID-19, and 13 (7.8%) related to personal protective equipment (PPE). Malfunctions were clinically significant in 61 (36.5% of 167) COVID-19 Treatment reports.

#### COVID-19 Tests

The 122 (73.1% of 167) reports about COVID-19 tests describe three different issues: the validity of testing, discomfort in the nostril after testing, and the tip of the swab breaking off inside the patient’s nose during a test.

“…A customer reported three samples producing a target 2 positive result with a very low ct value, which then tested negative on repeat. The customer was suspicious of the original results and did not report out results for these three samples…”

#### Equipment Used in the Treatment of COVID-19 Patients

The 32 (19.2% of 167) COVID-19 Treatment reports describe the equipment used in treating COVID-19 patients and elaborated on useful information about how ventilators and other COVID-19-related equipment can malfunction.

“… *A COVID-positive patient in prone position on the ventilator was able to turn his head from one side to the other. When doing this he was able to self-extubate as the plastic arm of the tube holder snapped off the crossbar that was attached to the patient’s face, rendering the ett* [endotracheal tube] *free from any securing fixture*…”

#### PPE

The 13 (7.8% of 167) reports about PPE all related to masks, including both medical and nonmedical masks. Reports questioned the integrity of the mask (e.g., pieces breaking, gaps at nose and cheeks) or the PPE manufacturing company (e.g., misleading claims of FDA approval).

“…I opened 1 pack and the masks have many holes in them with a see-thru extremely thin barrier…I purchased these 6 masks because ** said they were FDA approved and they are not.”

### Reports Coded as Investigative Delay

Of the reports coded as Investigative Delay, delays in investigating malfunctioning implants were the most common (87.1%, 2,754/3,161). The most common delays were with dental (75.5%, 2,080/2,754), followed by breast (11.1%, 305/2,754), and neural stimulation implants (6.7%, 185/2,754). Malfunctions were clinically significant in 2,932 reports (92.8% of 3,161 total reports coded as Investigative Delay).

#### Stock Language Referencing FDA Guidelines

The 2,080 (75.5% of 3,161) dental implant reports appear to be due to a bulk submission of reports from a single manufacturer. The manufacturer narratives contain similar stock language that directly references the FDA guidance for reporting in a pandemic. Some manufacturers stated that they were storing the nonfatal serious injury data, intending to submit a follow-up report.

“Non-fatal serious injury stored due to COVID-19 pandemic in accordance with FDA guidance ‘postmarketing adverse event reporting for medical products and dietary supplements during a pandemic’ published march 2020…”

#### Implants and In-Person Patient Care

The 2,754 (87.1% of 3,161) implant reports posed a unique issue for manufacturers attempting to evaluate a malfunctioning device. When implants malfunction, patients may need to visit their doctor for an in-person evaluation or even receive an explantation procedure to remove the device. Reports describe patients’ reluctance to visit their doctor’s office due to preexisting conditions and fear of contracting COVID-19.

“… Now, due to COVID-19, we are reluctant to go to hospital to replace or remove. Managing with oral antibiotics and reducing dose…”

Many hospital facilities in lockdown did not accept patients for elective procedures, preventing implants from being removed. Multiple reports mentioned that COVID-19 halted implant removal procedures. This delay impacted the speed of the facility and manufacturer investigations.

“…The patient was scheduled for a revision, however, the surgery was deemed non-essential and was cancelled due to COVID-19…”

### Implants Reaching the Patient

The dental implant reports made up more than half of the total reports in the dataset (59.4%, 2,080/3,500), and all of the reports described events that were clinically significant. All of the dental implant reports were classified as clinically significant because the dental implants did not perform as expected (i.e., nonosseointegration), requiring them to be explantated. Some reports also included infection at the implant site.

“…As per complaint ** after a clinical procedure, a dental implant displayed a failure of osseointegration and the implant was explanted.”

During the pandemic, some explantation procedures were canceled or delayed. The delay of an explantation procedure can have many different effects on the patient’s health depending on the type of implant, the severity of the malfunction, and the patient’s overall health. Several reports emphasized that the lack of an explantation procedure placed the patient in an unsafe situation that could lead to future harm.

”…The patient’s explantation surgery was delayed because of COVID-19 restrictions. Because of this, the patient’s right tissue expander was implanted in the patient for longer than 6 months, which is contraindicated in the IFU…”

Reports describe patients with malfunctioning implants who consequently no longer received symptom relief. The options for fixing the implants, and getting the patient’s treatment plan back on course during the lockdown, were few and far between.

“…The patient reported that she fell on an icy walkway three weeks ago and since her INS has not been working for her symptom control. She has been extremely happy with her symptom so the lack of symptom control made it pretty obvious that something was wrong. She went to the healthcare provider today and had two impedance checks done and both came up with black across the screen. She is scheduled for a lead revision after the COVID-19 is over…”

Reports also describe difficult situations where an implant harmed patients. In one such case, the patient believed they could not receive care due to the moratorium on elective procedures.

“…I now have fluid surrounding the implant, and capsule contracture. Along with a large list of symptoms. Rash, swelling, soreness and also enlarged thyroid gland. Waiting on more test results. Unable to get in for MRI and needle biopsy. Covid virus has all hospitals locked down…”

#### Travel Bans

Other manufacturers who mentioned the pandemic described system issues that prevented investigation into device failure. In one instance, the report described dispatching a service engineer to evaluate a malfunctioning device. However, the service engineer could not travel to the user facility to evaluate the device due to travel restrictions.

“… A field service engineer was dispatched but due to the lockdown in France because of the COVID-19 virus the unit will be inspected at a later date...”

#### Limited Capabilities of Manufacturers

Some manufacturers referenced halted investigations due to limited capabilities, though the reports do not thoroughly describe the cause of the limited capacity (e.g., employee absenteeism, stay-at-home orders).

“Due to the COVID-19 pandemic, the investigation for this event will be delayed. We will however, send the follow-up report as soon as our laboratory services are able to return to normal.”

#### Shipment of Devices

Reports mentioned devices not being returned to manufacturers, which required manufacturers to evaluate the devices and related events from the available information, such as device history or a report by a user facility. Some manufacturers decided not to accept devices for analysis at all to mitigate employee exposure to COVID-19.

“Following WHO declaration of a global health emergency situation due to the outbreak of corona virus sars-cov-2, it was decided to refrain from shipping of samples to support limiting the spread of the virus. In order to adapt to the global situation in the best possible way. The samples have not been investigated…”

## Discussion

Our analysis attempted to understand how the COVID-19 pandemic impacted medical device reporting by analyzing reports in the MAUDE database that mention COVID-19. From reports between January 1, 2020, and July 31, 2020, that mention COVID-19, 4.8% were coded as COVID-19 Treatment and described the medical devices used in the treatment, diagnosis, and prevention of COVID-19. In comparison, 90.3% of these reports were coded as Investigative Delay and describe the barriers manufacturers face while investigating malfunctioning devices during the pandemic.

The themes found in the reports are aligned with trends appearing elsewhere in healthcare during the pandemic. Research suggests that approximately 41% of Americans avoided or delayed medical care due to the pandemic.^8^ Additionally, elective procedures deferred in the summer of 2020 have created a surgical backlog across the world.^9-14^ One question after the pandemic is how patients, including those impacted by malfunctioning devices, will be given the care they need. Individual facilities will likely bear the responsibility of scheduling missed appointments and procedures. As cases fall, some patients will likely schedule necessary appointments on their own. However, a proactive approach would see facilities reaching out to their patients to encourage them to reschedule missed procedures, appointments, and yearly exams.

### Challenges With Investigating Malfunctions

For some manufacturers, the pandemic made investigating malfunctioning devices more challenging compared to their standard approach. Implants were uniquely impacted because a doctor’s visit or procedure is sometimes required to alter or replace an implant, and pandemic prevention measures emphasized limiting person-to-person contact. Reports described patients, especially those with increased risk factors for developing severe cases of COVID-19, who were hesitant to meet with their doctor in person. Simultaneously, governments and healthcare facilities canceled elective procedures, preventing implants from being replaced or extracted. This confluence of events created an environment where patients were not receiving medical care, and malfunctioning implants were particularly vulnerable to investigative neglect.

Investigators may not have been able to investigate devices thoroughly and may lose data about the malfunction over time. In the FDA’s guidance document, the government agency stipulates that manufacturers submit reports “within six months of the restoration of the adverse event reporting process to their pre-pandemic state.”^6^ Although it is unclear precisely what this timeframe means, it does suggest that manufacturers will likely be delaying investigations for, at a minimum, several months. Such a long delay brings into question how manufacturers will investigate a backlog of uninvestigated devices and whether those investigations will be fruitful after such a long hiatus. Additionally, because many of these reports were unable to be thoroughly investigated, preventable adverse events may have occurred and may continue to occur due to a lack of postmarket surveillance.

### Backlog of Uninvestigated Devices

It is important that manufacturers and the FDA address the backlog of uninvestigated devices so that unsafe devices can be identified and removed from the market. Additional guidance from the FDA may be necessary to guide the manufacturer investigations of previously uninvestigated reports. The guidance could include a system for prioritizing which report and device investigations should take priority moving forward and how to follow up with patients who were impacted by the malfunctioning device. Furthermore, future research should investigate strategies that manufacturers used to successfully investigate devices during the pandemic to provide examples of how postmarket surveillance can be resilient during crises in the future.

### Limitations

A limitation to this study is that medical device reporting is historically low. In 1996, as few as 0.5% of medical device errors were reported.^15^ This dataset does not represent all reasons manufacturers may delay an investigation and we cannot tell how many medical device reports remain uninvestigated. The counts of reports identified in the database do not correspond to the counts of events in the world.

Consequently, certain conclusions cannot be made based on this dataset. For example, the large number of dental implant reports does not indicate that dental implants are causing a disproportionate number of adverse events. Because our research is limited to reports with COVID-related phrases, and not all dental implants, we cannot make any conclusions about dental implants aside from the manufacturers’ use of language in their reporting. Comparing report numbers year to year should also be avoided as a fluctuation in the number of reports submitted to MAUDE can be based on a myriad of different factors from decreased reporting, improved device usability, changes to individual manufacturer and facility reporting strategies, and the pandemic.

## Conclusion

Aspects of the pandemic, such as travel bans and shipping policies, made it difficult for some manufacturers to investigate devices via traditional means. The nature of some devices, such as implants, also made facility and manufacturer investigations more challenging to perform. This change is a window into the novel patient safety issues arising in response to the pandemic. The lack of full-scale investigations into malfunctioning devices may lead to safety issues going unaddressed and harming additional patients in the future. It is currently unclear how uninvestigated malfunctions will be addressed after the pandemic.

## Figures and Tables

**Table 1.** Codebook for the Analysis of COVID-19-Related Reports

Relevance to COVID-19	
Code	Definition
**COVID-19 Treatment**	Reports that discuss devices and equipment directly used to diagnose, treat, and prevent COVID-19, its symptoms, and its spread. These included COVID-19 tests, ventilators, endotracheal tubes, feeding tubes, and personal protective equipment (PPE).

**Investigative Delay**	Reports that discuss delays to manufacturer’s investigation of a device in any MAUDE report due to COVID-19.

**Not Related**	Reports that mention COVID-19 incidentally, without reference to investigative delays or COVID-19 treatment.
**Patient Care Impact**	
**Clinically Significant**	Reports that describe medical device malfunctions that led to treatment changes, including receiving too little or too much of a drug; a follow-up procedure; injury; or death.

**Not Clinically Significant**	Reports that describe medical device malfunctions that did not change the patient’s treatment or condition.

**Table 2.** Frequency of COVID-19 Treatment and Investigative Delay MAUDE Reports Between January 1, 2020, and July 31, 2020

Relevance Categories	Clinically Significant	Not Clinically Significant	Total
**COVID-19 Treatment**	68	99	167
COVID-19 test	41	81	122
Equipment to treat COVID-19	25	7	32
PPE	2	11	13
**Investigative Delay**	2,936	225	3,161
Implant	2,748	6	2,754
*Dental*	*2,080*	*0*	*2,080*
*Breast*	*304*	*1*	*305*
*Neural Stimulator*	*185*	*0*	*185*
*Cardiac*	*75*	*2*	*77*
*Other*	*104*	*3*	*107*
Glucose Monitoring	37	109	146
External Infusion Pump	20	28	48
Catheter	29	9	38
Stapler	5	6	11
Other	97	67	164
Not Related	NA	NA	172
Total	3,004	324	3,500
